# Examination of Endogenous Rotund Expression and Function in Developing *Drosophila* Olfactory System Using CRISPR-Cas9–Mediated Protein Tagging

**DOI:** 10.1534/g3.115.021857

**Published:** 2015-10-23

**Authors:** Qingyun Li, Scott Barish, Sumie Okuwa, Pelin C. Volkan

**Affiliations:** * Department of Biology, Duke University, Durham, North Carolina 27708; † Duke Institute for Brain Sciences, Duke University, Durham, North Carolina 27708

**Keywords:** CRISPR, Cas9, tagging, homologous recombination, genome editing, rotund, olfactory system development

## Abstract

The zinc-finger protein Rotund (Rn) plays a critical role in controlling the development of the fly olfactory system. However, little is known about its molecular function *in vivo*. Here, we added protein tags to the *rn* locus using CRISPR-Cas9 technology in *Drosophila* to investigate its subcellular localization and the genes that it regulates . We previously used a reporter construct to show that *rn* is expressed in a subset of olfactory receptor neuron (ORN) precursors and it is required for the diversification of ORN fates. Here, we show that tagged endogenous Rn protein is functional based on the analysis of ORN phenotypes. Using this method, we also mapped the expression pattern of the endogenous isoform-specific tags *in vivo* with increased precision. Comparison of the Rn expression pattern from this study with previously published results using GAL4 reporters showed that Rn is mainly present in early steps in antennal disc patterning, but not in pupal stages when ORNs are born. Finally, using chromatin immunoprecipitation, we showed a direct binding of Rotund to a previously identified regulatory element upstream of the *bric-a-brac* gene locus in the developing antennal disc.

The *rotund* (*rn*) gene is a critical component of developmental programs in the fly. It has been shown to function in the development of the eye, leg, and antenna (Baanannou *et al.* 2013; Li *et al.* 2013; Pueyo and Couso 2008; St Pierre *et al.* 2002; Terriente Félix *et al.* 2007). We have previously reported that Rn functions in the developing antenna to increase the amount of neuronal diversity in the olfactory system by a factor of two (Li *et al.* 2013). The timing of *rn* expression slightly overlaps with that of olfactory receptor (OR) genes, suggesting that Rn may bind directly to OR promoters. Although we demonstrated that Rn is a regulator of OR expression, we were not able to detect binding to OR promoters with *in vitro* assays (Li *et al.* 2013). Based on sequence analyses, Rn is predicted to be a transcription factor, and other groups have shown that Rn binds to a T-rich motif *in vitro* (Baanannou *et al.* 2013). Investigations of *rn* expression have, up to this point, relied on RNA *in situ* and reporters (Li *et al.* 2013; St Pierre *et al.* 2002), and no visualization of Rn protein has been made on its expression in the developing olfactory system. The subcellular localization of Rn protein also remains unknown. Understanding the molecular function of Rn may yield critical insights into the processes of olfactory receptor neuron (ORN) differentiation and diversity.

Attempts to raise antibodies against Rn have thus far been unsuccessful, and we therefore chose to tag Rn in its endogenous locus. Recent advances in CRISPR-Cas9 technology have made it an attractive method for genome editing due to its speed, ease of use, and high success rate. Here, we report the incorporation of a protein tag to the *rn* gene locus without disrupting its function. Using this tagged protein, we were able to examine the expression pattern of the functionally relevant isoforms of the endogenous Rn protein, and we detected temporal differences in the duration of gene expression compared to previously published results. In addition, we found that Rn is localized to the nucleus, where it is excluded from the DAPI-dense heterochromatic region. Finally, we showed that *bric-a-brac* (*bab*) locus is a direct target of Rn *in vivo* using chromatin immunoprecipitation (ChIP). These results provide new clues about Rn molecular functions and the mechanisms of action during ORN diversification.

## Materials and Methods

**Figure 1:**
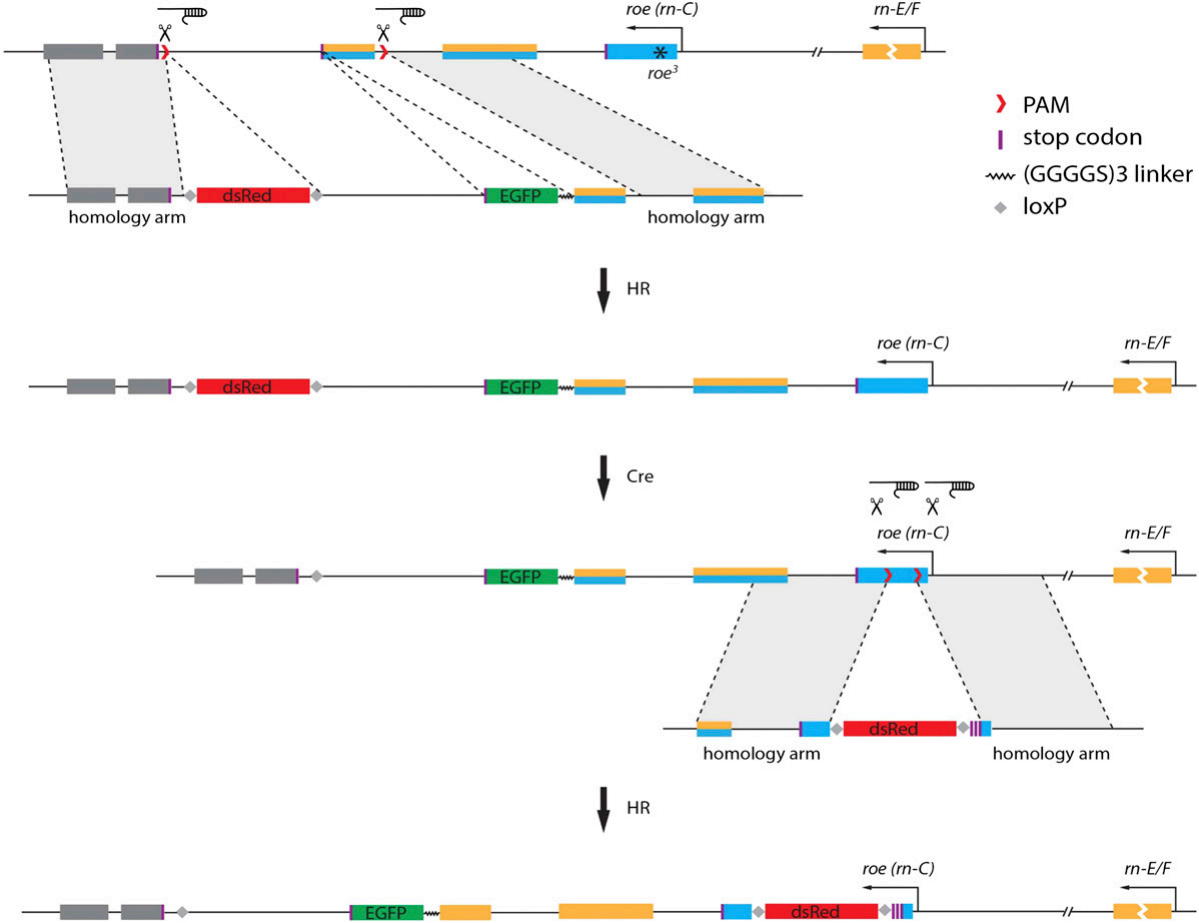
Molecular cloning design for CRISPR-induced tagging repair templates. The *rn* locus on the genome is oriented from right (5′) to left (3′). E/F (yellow) and C (blue) isoforms share the last two exons. Introns and intergenic regions are shown as a line. Individual elements are depicted in the scheme. All homology arms (in gray shade) are approximately 1 kb. Only the construct for EGFP-tagging is shown. The 3XFLAG tagging scheme is identical except for the tag. To generate isoform-specific tagging, the *roe^3^* allele was used in one of the two approaches and is indicated by an asterisk. HR, homologous recombination.

**Figure 2:**
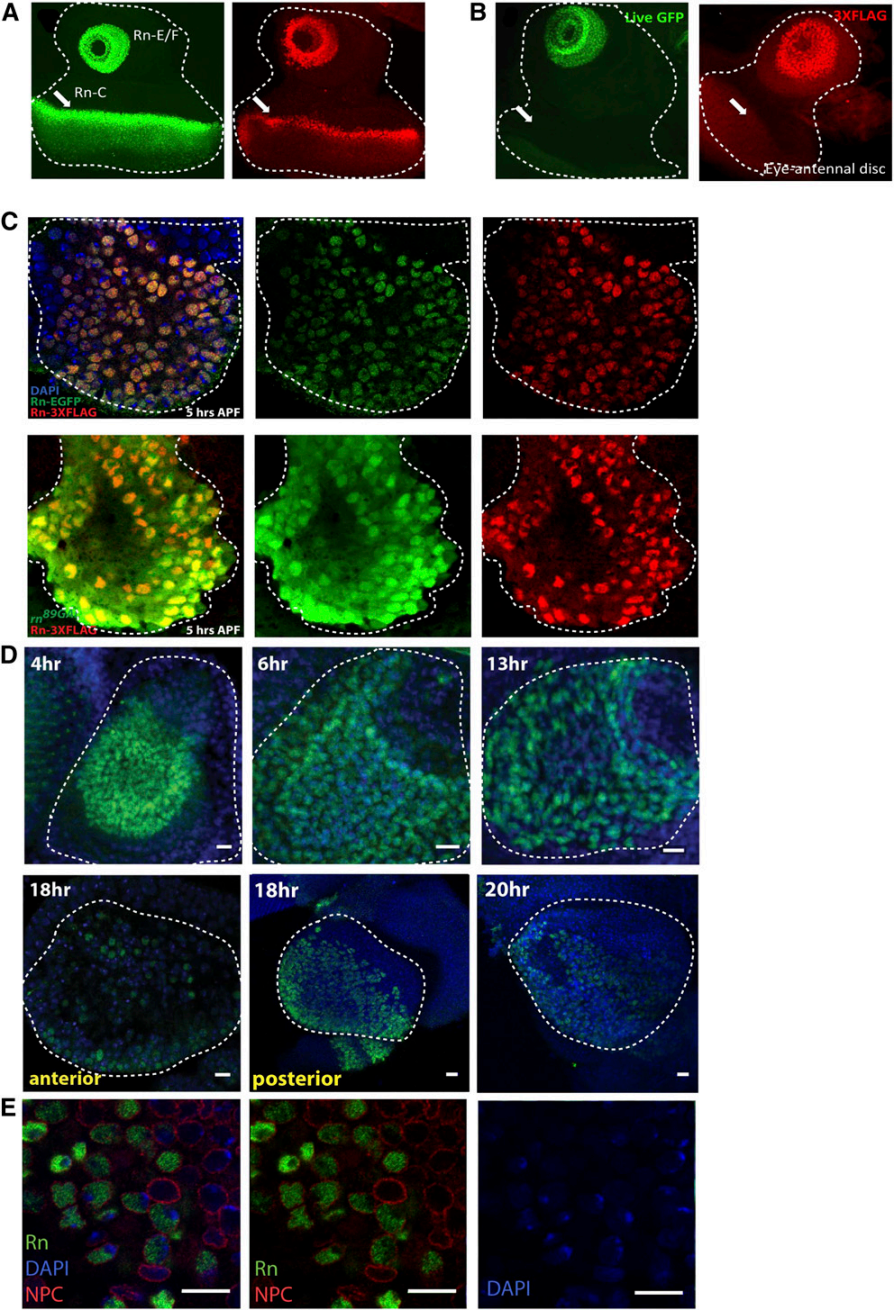
Expression of Rn tagged constructs. (A) Expression of EGFP (green) and 3X-FLAG (red) tagged Rn constructs in third instar larval eye antennal discs. Both constructs label all Rn isoforms and, correspondingly, expression is observed in both the antennal disc and the morphogenetic furrow of the eye disc. (B) Expression of E/F-specific tagged Rn constructs. Expression in the morphogenetic furrow of the eye disc is lost in isoform-specific tagging (arrow). (C) Staining for Rn constructs in antennal discs at 5 hr APF. Both Rn-EGFP (green) and Rn-3XFLAG (red) constructs exactly overlap and also overlap with the E/F-specific *rn^89GAL4^* reporter. (D) Time course of E/F-specific Rn-EGFP expression. Rn is expressed in the developing antennal disc in a ring pattern from 4 to 13 hr APF. By 18 hr APF, Rn expression as the antenna forms begins to decrease, with the exception of a lateral region in and near the arista. By 20 hr APF, Rn expression is nearly entirely absent in the developing antenna. Dashed lines outline antennal disc or developing third antennal segment in (A) to (D). (E) Rn protein is localized to the nucleus. Costaining for DAPI (blue) and the nuclear pore complex (NPC) (red) reveals that Rn (green) is present in the nucleus and is excluded from DAPI dense heterochromatic regions. All scale bars represent 10 μm.

The *rn* gene locus encodes three isoforms, but only the E/F isoforms are relevant to ORN diversification (Li *et al.* 2013; St Pierre *et al.* 2002). Because all three isoforms share the same 3′ end region, we decided to add an EGFP or 3XFLAG tag to this end, which would label all three transcripts potentially without disrupting any isoform function by the insertion of the tag (Figure 1). Two CRISPR cutting sites were induced by the germline-specific Cas9 transgene under the guidance of two separate chimeric RNAs (chiRNAs). The chiRNAs were transcribed from the injected plasmid constructs. Once the cuts were made, the sequence between the cutting sites containing the last common exon and the 3′ UTR region was replaced with an exogenously engineered sequence supplied as a double-stranded DNA repair template via homologous recombination (*HR*) (Figure 1). One-kb homology arms flank each cutting site. The newly incorporated sequence contains the same exon–stop codon removed–connected to a tag via a flexible linker. In addition, it contains a DsRed selectable marker flanked by loxP sites, which facilitates genetic screening (Figure 1) (Gratz *et al.* 2014; Horn *et al.* 2000). Through this manipulation, we were able to obtain EGFP or 3XFLAG tagged Rn stocks, which we named Rn-EGFP and Rn-3XFLAG, respectively (Figure 2A).

### Guide chiRNA Design

To tag all three *rn* isoforms (C, E, and F) with EGFP or 3XFLAG at the common 3′ end, two cutting sites were selected, one in the last intron (referred to as “intron”) and the other in the intergenic region downstream of *rn* coding region (referred to as “inter”) (Figure 1). Twenty base-pair long target sites were selected using the flyCRISPR Optimal Target Finder tool on the flyCRISPR website (Gratz *et al.* 2014). We set the parameter for initial nucleotide selection as “All CRISPR targets” and would simply add a G at the beginning, if it does not include one, to facilitate its transcription by the U6 promoter (as seen by the addition of g/c for the intergenic target we used in Table 1, ID: 3, 4). We then evaluated potential off-targets for all the candidates by setting “Maximum” for “stringency” and “NGG and NAG” for “PAM.” The sites chosen as candidates with minimal potential off-targets were confirmed to have no mutations in the fly stocks in which we would inject (Table 2). Such a site from each of the “intron” and “inter” regions was cloned into the pU6-BbsI-chiRNA plasmid following the U6-gRNA (chiRNA) cloning protocol on the same website. The oligos used to generate the chimeric guide RNAs can be found in Table 1 (ID: 1–4). The final constructs are named rn-chiRNA-intron and rn-chiRNA-inter.

**Table 1:** CRISPR oligos

**Primer ID**	**Name**	**Sequence**
1	rn-chiRNA-intron-sense	cttcGTTGTGAAGAATCGAAGAGA
2	rn-chiRNA-intron-antisense	aaacTCTCTTCGATTCTTCACAAC
3	rn-chiRNA-inter-sense	cttcgATATTCCGAGACACAGGGGA
4	rn-chiRNA-inter-antisense	aaacTCCCCTGTGTCTCGGAATATc
5	5HDR-rnCR-F	ggtaccCATCAGCGCAACAACCTGG
6	5HDR-rnCR-R	ctcgagTCCCTTGTCCTTCCCAGGA
7	EGFP-cDNA-N	ctcgagATGGTGAGCAAGGGCGAG
8	EGFP-cDNA-C	gtcgacTTACTTGTACAGCTCGTCCATGC
9	3G4S-Xhol-F	tcgagGGAGGAGGCGGCTCCGGAGGCGGAGGATCCGGCGGAGGTGGCTCCc
10	3G4S-Xhol-R	tcgagGGAGCCACCTCCGCCGGATCCTCCGCCTCCGGAGCCGCCTCCTCCc
11	3G4S-3XFLAG-F	tcgagGGAGGAGGCGGCTCCGGAGGCGGAGGATCCGGCGGAGGTGGCTCCGACTACAAAGACCATGACGGTGATTATAAAGATCATGACATCGATTACAAGGATGACGATGACAAGTAAg
12	3G4S-3XFLAG-R	tcgacTTACTTGTCATCGTCATCCTTGTAATCGATGTCATGATCTTTATAATCACCGTCATGGTCTTTGTAGTCGGAGCCACCTCCGCCGGATCCTCCGCCTCCGGAGCCGCCTCCTCCc
13	3HDR-rnCR-F	gcggccgCCTGTGTCTCGGAATATCATTTTGG
14	3HDR-rnCR-R	gagctcATTGCAAGGGGTCTGAACTG
15	3UTR-rnCR-F	gtcgacCTAGGGGCCTACTTCTAGATGG
16	3UTR-rnCR-R	aagcttGGAAGGATAACATTTAATTTACTTTATTACG
17	rnC-chiRNA-5′-F	cttcGGCGGAATCTCCCCAATCAG
18	rnC-chiRNA-5′-R	aaacCTGATTGGGGAGATTCCGCC
19	rnC-chiRNA-3′-F	cttcGATCCGGGACTTGCGGCCCC
20	rnC-chiRNA-3′-R	aaacGGGGCCGCAAGTCCCGGATC
21	rnC-5HDR-Kpnl-F	ggtaccATGTCTGCGCCTGAATGACT
22	rnC-5HDR-Spel-R	actagttaattagttaCAGCGGCGAGTTGTGATGGTAG
23	rnC-3HDR-Notl-F	gcgGCCGCAAGTCCCGGATCTAC
24	rnC-3HDR-Sacl-R	gagctcGATGCCTGCACTTGTACGG

All restriction enzyme sites for cloning are underlined. The nucleotide labeled in red from ID 3 or 4 is the addition of g/c to aid transcription by the U6 promoter. The sequence labeled in red from ID 11 or 12 encodes 3XFLAG. The four triplets labeled in red from ID 22 are the added stop codons.

**Table 2:** CRISPR injection scheme

**Injected Flies**	**Donor**	**chiRNA 1**	**chiRNA 2**
vas-Cas9.RFP(−) (/FM7a,Tb)	rnCR-EGFP	rn-chiRNA-intron	rn-chiRNA-inter
vas-Cas9.RFP(−) (/FM7a, Tb)	rnCR-FLAG	rn-chiRNA-intron	rn-chiRNA-inter
vas-Cas9.RFP(−); roe[3]/TM3	rnCR-EGFP	rn-chiRNA-intron	rn-chiRNA-inter
vas-Cas9.RFP(−); roe[3]/TM3	rnCR-FLAG	rn-chiRNA-intron	rn-chiRNA-inter
vas-Cas9.RFP(−); rn-EGFP DsRed(−)	rnC-STOP	rnC-chiRNA-5′	rnC-chiRNA-3′

vas-Cas9.RFP(−) chromosome (FBst0055821) is from Bloomington stock #55821; roe[3] chromosome (FBst0007411) is from #7411. The majority of the injected embryos from the first and second experiments are homozygous for vas-Cas9.RFP- (thus parentheses for FM7a); all the rest are homozygous for vas-Cas9.RFP(−).

### Repair template design

To make the repair template plasmid, we created ∼1-kb homology arms for both cutting sites. Specifically, the fragment that includes the homology arm upstream of the “intron” cutting site is named 5HDR (5′ homology-directed repair). It contains a 1041-bp homology arm and the following 333 bp right before the stop codon. The fragment was amplified using primers listed in Table 1 (ID: 5, 6) as a single piece of DNA from fly stocks used for injections (Table 2). *Kpn*I and *Xho*I cutting sites were added to allow for cloning into the pBluescript II SK vector.

Importantly, to protect this repair fragment from being cut by rn-chiRNA-intron, we mutated three nucleotides in the CRISPR recognition site using a Site-Directed Mutagenesis Kit (Agilent Technologies) so that it changed from gttgtgaagaatcgaag | agaCGG to gttgtgaagaatagaga | agaCGG (PAM is capitalized; the presumable cutting site is denoted as “|”; mutated nucleotides are underlined). This mutation should not affect homologous recombination because they were not part of the homology arm and would only replace the sequence that we intended to remove. In addition, because these mutations are immediate to the cutting site, they should effectively block chiRNA recognition, thereby protecting the template. We also made sure the changes would not affect splicing signals due to its proximity to the acceptor site.

To make the EGFP tag, the coding region of EGFP with the start and stop codons was amplified from the pTGW vector flanked by *Xho*I and *Sal*I sites (primers are in Table 1, ID: 7, 8). A flexible (GGGGS)3 linker was incorporated between the 5HDR fragment and EGFP to facilitate protein folding. To do this, 5′ phosphorylated oligos (Table 1, ID: 9, 10) with *Xho*I overhangs were synthesized and annealed. The same linker was added between the 3XFLAG tag and 5HDR, except that the 3XFLAG coding sequence and the linker were synthesized as a single fragment flanked by *Xho*I overhangs. These two 5′ phosphorylated oligos were annealed. The 3XFLAG coding region was optimized for fly codon usage (see Table 1, ID: 11, 12, for sequences). For the homology arm downsteam of the intergenic cutting site, a 1388-bp fragment, named 3HDR, immediately starting from the cutting site was amplified (primers are in Table 1, ID: 13, 14). It was flanked by *Not*I and *Sac*I sites. The leftover sequence between the 5HDR and 3HDR fragments (mainly 3′ UTR of *rn*) was cloned as it is with *Sal*I and *Hin*dIII on either side (primers are in Table 1, ID: 15, 16).

To aid with the genetic screening process, we added a 3XP3 DsRed selectable marker, which can eventually be excised from the genome by the expression of Cre protein (Gratz *et al.* 2014; Horn *et al.* 2000).

This cassette was obtained by cutting the pHD-DsRed-attP vector with *Spe*I and *Not*I restriction enzymes. All six fragments for the Rn-EGFP injection (or five fragments for Rn-3XFLAG) were sequentially inserted into the pBluescript II SK vector (Figure 1). The final constructs are named pBS-RnEGFP-crHDR_Cas9 (rnCR-EGFP for short) and pBS-Rn3XFLAG-crHDR_Cas9 (rnCR-FLAG for short). They were fully sequenced before being injected into the vas-Cas9.RFP(−)/FM7a embryos, which have a wild-type third chromosome (Table 2). Also, note that *rn*: FBgn0267337 is on the third chromosome. These two injections yielded Rn-EGFP and Rn-3XFLAG lines, in which all three isoforms are tagged (Figure 2A). We then crossed these two lines to *hs*-Cre without heat shock to remove the DsRed cassette from the genome.

### Isoform-specific tagging

We also attempted to make an E/F isoform-specific tagging line by injecting the same chiRNAs and repair template (except relevant fragments being amplified from *roe^3^* larvae) mixture into the *roe^3^* heterozygous stock. Because *roe^3^* harbors an amber stop codon in the first specific exon of the C isoform (St Pierre *et al.* 2002), a successful recombination onto the mutant chromosome would lead to a truncated C isoform without the tag and full-length E/F isoforms with the tag (Figure 1). As expected, we obtained the Rn-E/F isoform-specific 3XFLAG tagging line from the screening, which was named RnE/F-3XFLAG (Figure 2B). However, the EGFP tagging yielded no correct targeting event, presumably due to the heterozygous background, a larger tag compared to 3XFLAG, and very low survival/fertility rates.

To obtain the EGFP-tagged Rn specific to the E/F isoforms, we decided to conduct a second round of CRISPR-mediated editing in the Rn-EGFP line to insert stop codons at the beginning of the first C isoform-specific exon. This way, only the E/F isoforms, and not the C isoform, would be fully translated with the tag. Because, unlike the *roe^3^* over a balancer background, the Rn-EGFP line is healthy when homozygous and a targeting event occurring to either chromosome is sufficient for our purpose, we expect higher chances of obtaining the target line. This second trial yielded ∼50% germline transmission efficiency, giving rise to the RnE/F-EGFP line (Figure 2B, Table 3).

**Table 3:** CRISPR screening results

**ID**	**Injected Flies***^a^*	**Donor**	**Larvae**	**Founder Fertility***^b^*	
				Male	Female
1	vas-Cas9.RFP(−) (/FM7a,Tb)	rnCR-EGFP	173	20(2)/44(3)	22(6)/59(10)
2	vas-Cas9.RFP(−) (/FM7a,Tb)	rnCR-FLAG	151	17(2)/49(3)	22(4)/37(6)
3	vas-Cas9.RFP(−); roe[3]/TM3	rnCR-EGFP	188	8/31	18/42
4	vas-Cas9.RFP(−); roe[3]/TM3	rnCR-FLAG	158	3/43	16/40
5	vas-Cas9.RFP(−); rn-EGFP DsRed(−)	rnC-STOP	174	21/60	15/64

**Founders Yielding DsRed+ G1***^c^*		**Founders with DsRed+ Offspring Yielding Targeted Tagging Event***^d^*		**Overall Germline Transmission***^e^*	
Male	Female	Male	Female	Male	Female
9 (2)/20 (2)	2/22 (6)	3 (1)/9 (2)	1/2	3 (1)/20 (2); 15.0%	1/22 (6); 4.5%
6 (1)/17 (2)	4/22 (4)	4 (1)/6 (1)	3/4	4 (1)/17 (2); 23.5%	3/22 (4); 13.6%
1/8	1/18	0/1	0/1	0/8; 0%	0/18; 0%
0/3	3/16	0/0	1/3	0/3; 0%	1/16; 6.3%
13/21	7/15	11/13	7/7	11/21; 52.4%	7/15; 46.7%

a vas-Cas9.RFP(−) chromosome is from Bloomington stock #55821; roe[3] chromosome is from #7411. The majority of the injected embryos from ID 1 and 2 are homozygous for vas-Cas9.RFP(−); all the rest are homozygous for vas-Cas9.RFP(−).

b The format is the number of fertile flies over the number of survived adult flies. The numbers in parentheses are for founders of vas-Cas9.RFP(−)/FM7a,Tb genotype.

c The format is the number of flies in the category over the number of fertile flies. The numbers in parentheses are for founders of vas-Cas9.RFP(−)/FM7a,Tb genotype. At least 60 or all G1 flies from each fertile founder were screened for DsRed.

d The format is the number of flies in the category over the number of founders yielding DsRed+ G1. The numbers in parentheses are for founders of vas-Cas9.RFP(−)/FM7a,Tb genotype. At least 60 or all G1 flies from each fertile founder were screened for DsRed, and approximately 10 individual DsRed+ G1 (unless fewer flies were recovered, which would all be used) from each candidate founder were crossed. For ID 1 and 2, all fertile DsRed+ G1 were PCR-screened and stained to test the presence of tags. For each founder lineage, at least one G1 fly with positive results for all the tests was sequenced for the targeted region to confirm a clean homologous recombination event. For ID 3, 4, and 5, G2 larvae were stained to check the tags before sequencing to confirm targeted events. Potential off-targets regions were PCR-amplified for sequencing.

e The percentage is calculated as the proportion of fertile founders that yield targeted tagging events. The numbers in parentheses are for founders of vas-Cas9.RFP(−)/FM7a,Tb genotype.

To add these early stop codons, two cutting sites were selected in the first exon for the C isoform using the same criteria during all isoform tagging designs, and the oligos were annealed and cloned as described above. The oligos used can be found in Table 1 (ID: 17-20). These constructs are named rnC-chiRNA-5′ and rnC-chiRNA-3′.

We used ∼1-kb homology arms, named rnC-5HDR and rnC-3HDR, flanking the cutting sites. For amplifying the rnC-5HDR fragment, a 10-bp sequence between the *Spe*I cloning site and 5′ end of the cut leftover was included in the reverse primer. Along with *Spe*I site, this 16-bp fragment encodes four stop codons in all three frames, including two stop codons in the frame that we intended to manipulate (Table 1, ID: 21, 22). This homology arm is flanked by *Kpn*I and *Spe*I. To make the rnC-3HDR, a 1148-bp fragment flanked by *Not*I and *Sac*I was amplified using primers from Table 1 (ID: 23, 24).

Finally, the sequences on both sides of 3XP3 DsRed from the rnCR-EGFP construct (described above) were replaced by rnC-5HDR and rnC-3HDR using the *Kpn*I/*Spe*I and *Not*I/*Sac*I sites, respectively. This yields the rnC-STOP construct, which was used as the repair template to be injected into the vas-Cas9.RFP(−); Rn-EGFP DsRed(−) line. Because the 3XP3 DsRed cassette was excised from the Rn-EGFP flies after initial screening, it was possible to reuse 3XP3 DsRed cassette as a selectable marker for efficient genetic screening.

### Embryo injection

The injection mixtures were prepared as instructed on the flyCRISPR website. Specifically, 100 ng/µL each of pU6-BbsI-chiRNA and 500 ng/µL repair template were mixed and injected into ∼200 embryos for each manipulation following a standard protocol (Table 2).

### Targeted event screening

Single G_0_ founders were crossed to balancer flies, and G1 flies were screened for the presence of 3XP3 DsRed. For each founder line that yielded DsRed (+) progeny, ∼10 individual G1 flies were crossed to the balancer stock, unless fewer flies were produced. DNA from G2 larvae or individual G1 adult flies was extracted and PCR was screened for the presence of EGFP/3XFLAG. G2 larvae were dissected to check for live GFP (or stained with FLAG antibody) in the eye-antennal discs. The targeted area was sequenced for the G1 lineages that show positive PCR results and EGFP/FLAG signals to confirm that the targeted event is 100% correct. Candidate nonspecific targeting sites reported by the CRISPR target finder tool were sequenced for potential off-targets. A couple of healthy G2 lines that passed all these tests were crossed to the *hs*-Cre line to excise the 3XP3 DsRed cassette, from which we obtained the lines with Rn tagged by EGFP or 3XFLAG in the endogenous locus (Figure 2A).

Consistent with previous reports (Bassett and Liu 2014), from CRISPR screenings we did here, founder lines have relatively low survival (55.6% on average) and fertility (34.5% of survived on average) rates. Nonetheless, within the lines that are fertile, the overall germline transmission rate is 18.5% on average, which is expected for a common sized screen (Table 3).

### Chromatin immunoprecipitation

ChIP procedure is modified from a previous protocol (Slattery *et al.* 2011). For each genotype, approximately 800 eye-antennal discs were dissected. The samples were cross-linked with 1% formaldehyde in dissection buffer for 10 min at room temperature. To quench cross-linking, glycine was added to 125 mM final concentration, and the samples were incubated for 5 min. The discs were homogenized and sonicated in a Bioruptor machine for 13 min (high frequency; 30 sec ON/30 sec OFF). The chromatin was precleared with prewashed Dynabeads Protein G (Life Technologies) for 1 hr at 4° on a nutator. The precleared chromatin was split into two tubes (1 ml/tube), and another 20 μl (2%) was saved as input and stored at −20°; 5 μg anti-GFP antibody (Ab290) or an equal amount of normal rabbit IgG were added to either tube, followed by overnight incubation at 4°. Beads were added to both tubes, and the samples were incubated for 2 hr at 4° on a nutator. Beads were briefly rinsed with wash buffer I (50 mM K-HEPES, pH 7.8, 140 mM NaCl, 1 mM EDTA, 1 mM EGTA, 1% Triton X-100, 0.1% Na-deoxycholate, 0.1% SDS) and washed once with wash buffer I, once with wash buffer II (the same as buffer I, except that NaCl is 500 mM), once with wash buffer III (250 mM LiCl, 0.5% Igepal CA-630, 0.5% Na-deoxycholate, 1×TE), and twice with the TE buffer at 4° for 5 min/each wash. The chromatin was eluted twice with prewarmed elution buffer (1% SDS, 100 mM NaHCO_3_). For each elution, beads were incubated in 100 μl solution for 10 min at 65°, with gentle vortexing every 2–3 min. To reverse cross-link, 5 M NaCl was added to each tube, followed by overnight incubation at 65°. The ChIP-ed DNA was treated with RNase and proteinase K, and extracted by PCR purification columns (Qiagen). The purified DNA was tested for enrichment of DNA fragments by qPCR. To test direct binding of Rn to the published 13-bp T13 motif within the *bab2* LAE (leg and antennal enhancer) *in vivo*, a primer pair covering this region was designed for ChIP-qPCR analysis (Baananou *et al.*, 2013). To confirm that Rn does not bind to the M1 motif upstream of *rn*-positive OR promoters (Li *et al.* 2013), a primer set covering the motif of the Or82a promoter was designed and used in ChIP-qPCR analysis (Table 4).

**Table 4:** ChIP-qPCR primers

Primer Name	Sequence
Bab2_ChIP_T13_F	TATTTGCGTGGAGCCTTC
Bab2_ChIP_T13_R	TAACGATTGCCGCGATTT
Or82a_ChIP_M1_F	CACAGTACATACAGCCATACAG
Or82a_ChIP_M1_R	CGCTTCCTTCTGCTTGTT

### Quantitative RT-PCR

qRT-PCR was performed using the FastStart Universal SYBR Green Master Mix (Roche) or the FastStart Essential DNA Green Master Mix using standard protocol. Enrichment for each fragment was analyzed in triplicate.

### Data availability

All mentioned tagged lines are available upon request.

## Results and Discussion

### Tagging of Rn protein

The zinc-finger protein Rotund (Rn) was previously shown to be expressed in a subset of olfactory receptor neuron precursors in the third instar antennal discs, where it plays a critical role in neuronal diversification (Li *et al.* 2013). In the olfactory system, mutations in *rn* are associated with a loss of ORN classes originating from *rn*-positive precursors and an expansion of some default *rn-negative* identities. To identify the role of Rn in ORN precursor diversification, we wanted to identify tissue and subcellular localization of Rn in the developing olfactory system, in addition to identifying direct target genes regulated by Rn using chromatin immunoprecipitation. However, our attempt to raise antibodies against Rn in rabbits was unsuccessful. In addition, GAL4/UAS-mediated expression of *rn-E* cDNA failed to rescue the mutant phenotype and caused a dominant effect in the olfactory system (Li *et al.* 2013), which eliminated the option of tagging Rn in such a transgenic construct. We decided to use the CRISPR-Cas9 technology, which has been shown to work in flies as a simple way of editing the genome (Gratz *et al.* 2013; Yu *et al.* 2013; Yu *et al.* 2014; Kondo and Ueda 2013; Ren *et al.* 2013; Gratz *et al.* 2014), to insert an epitope tag fused with the Rn reading frame. The tagged Rn would likely behave more similarly to the wild-type protein under the control of endogenous gene regulatory apparatus, and this line can be subject to biochemical analysis using antibodies against the tag.

The *rn* gene locus encodes three isoforms, C, E, and F, all of which share a common last exon that contains zinc-finger domains, and we therefore chose to tag this exon with either EGFP or 3XFLAG. However, only the E and F isoforms are relevant to olfactory system development. To tag the E and F isoforms specifically, we created mutations that affect only the C isoform in our CRISPR lines (for a detailed description of tagging procedure see *Materials and Methods*).

### Isoform-specific tagging of Rn

To obtain the EGFP-tagged Rn specific to the E/F isoforms, we decided to conduct a second round of CRISPR-mediated editing in the Rn-EGFP line to insert stop codons at the beginning of the first C isoform-specific exon. This way, only the E/F isoforms, and not the C isoform, would be fully translated with the tag. Because the Rn-EGFP line is healthy when homozygous, and because a targeting event occurring to either chromosome is sufficient for our purpose, we expect higher chances of obtaining the target line. This second trial yielded ∼50% germline transmission efficiency, giving rise to the RnE/F-EGFP line (Figure 2B, Table 3, also see *Materials and Methods*).

### Testing the expression pattern of the tagged Rn protein

GAL4-dependent expression analysis in developing larval antennal disc and pupal antenna showed that Rn is expressed in a subset of ORN precursors in early pupal stages. Rn expression ceases after 40–50 hr after puparium formation (APF), which coincides with the onset of olfactory receptor expression, yet Rn does not bind to OR promoters (Li *et al.* 2013). Thus, endogenous Rn function and expression might be restricted to precursor specification rather than later stages where it functions to directly regulate OR genes. Many developmental expression analyses involve utilization of reporter expression assays, where the perdurance of an exogenous reporter gene can mask the normal expression dynamics of endogenous proteins. To test whether endogenous Rn expression and function are restricted to precursor stages prior to the onset of OR expression, we compared the expression pattern of tagged Rn protein in the developing olfactory system to previous studies on GAL4-mediated reporter expression. It has been shown that *rn*^89*GAL4*^ enhancer trap driven GFP expression recapitulates the endogenous RNA expression pattern *in situ* in third instar larval antennal discs (Li *et al.* 2013; St Pierre *et al.* 2002). These studies showed that Rn is expressed in the antennal disc in a ring pattern, and it persists in this ring through morphogenetic events that generate the antenna and is turned off approximately 50 hr after puparium formation (APF). It is important to note that *in situ* studies have not been conducted at pupal stages. Thus, the *rn^89GAL4^* reporter has not been validated at these stages in the antenna. During these processes, Rn is required to specify a subset of olfactory receptor neuron (ORN) precursors (Li *et al.* 2013). Developmental analysis of both Rn-EGFP and Rn-3XFLAG expression confirmed that expression of Rn is found in third instar antennal imaginal discs (Figure 2, A and B) and that it overlaps with the reporter expression (Figure 2C). However, unlike previous GAL4-based detection methods, which showed a downregulation of *rn* by 50 hr APF, tagged Rn expression is nearly absent by 20 hr APF (Figure 2D). These results suggest that tagged versions of Rn are more precise in reporting protein dynamics and the GAL4-driven GFP perdurance masks the temporal expression pattern of endogenous Rn protein. More importantly, this information acquired from the newly generated lines points to an earlier and narrower critical window requiring Rn function for ORN fate specification than expected before. Considering ORN precursor divisions are prominent during 16–22 hr APF (Sen *et al.* 2003) when the Rn protein level is already dramatically reduced, it is possible that the major function of Rn is only required during or prior to the precursor stage. In addition, because the OR expression occurs even later (around the mid-pupal stages), a direct regulation of OR genes by Rn becomes highly unlikely, even though it was suggested by the overlap of the two events from the previous expression analysis. The results from this study suggest that the ORN precursor specification step, which correlates with larval and early pupal stages, is essential for ORN diversity in the adults.

In the past, we were able to detect Rn-C isoform in the developing antenna by RT-PCR (Li *et al.* 2013). Mutagenesis of the Rn-C isoform using CRISPR-Cas9 leads to a visible loss of this expression in the eye portion as expected, but also reveals the true patterns of E and F isoform (Figure 2, A and B). This new tool would allow us to pinpoint the exact function of Rn protein and provide a handle for uncovering the molecular mechanism of ORN diversification.

### Subnuclear localization of Rn

Rn is a C2H2 zinc finger protein thought to function in the regulation of transcription (Baanannou *et al.* 2013; Li *et al.* 2013; St Pierre *et al.* 2002; Terriente Félix *et al.* 2007). However, its subcellular localization is not known. Subnuclear localization of transcription factors can give clues to the type of gene regulation. For example, some factors might exhibit punctate subnuclear expression, whereas others might exclusively be localized to heterochromatic or euchromatic regions. Given its function in transcriptional regulation, we predicted that Rn is localized to the nucleus. To determine this, we investigated the subnuclear localization of the endogenous tagged Rn protein. These analyses showed that Rn is specifically localized to the nucleus (Figure 2E). According to current knowledge, heterochromatic regions in cells are stained densely with DNA dyes such as DAPI or Hoechst due to an increased AT-rich DNA content and density (Zimmer and Wähnert 1986). Interestingly, DAPI staining together with antibodies against the nuclear pore complex also indicated that Rn expression is specifically excluded from DAPI dense regions that are associated with heterochromatin (Figure 2E). These results suggest that Rn is a nuclear protein excluded from heterochromatin.

### Testing functionality of the tagged Rn protein

**Figure 3:**
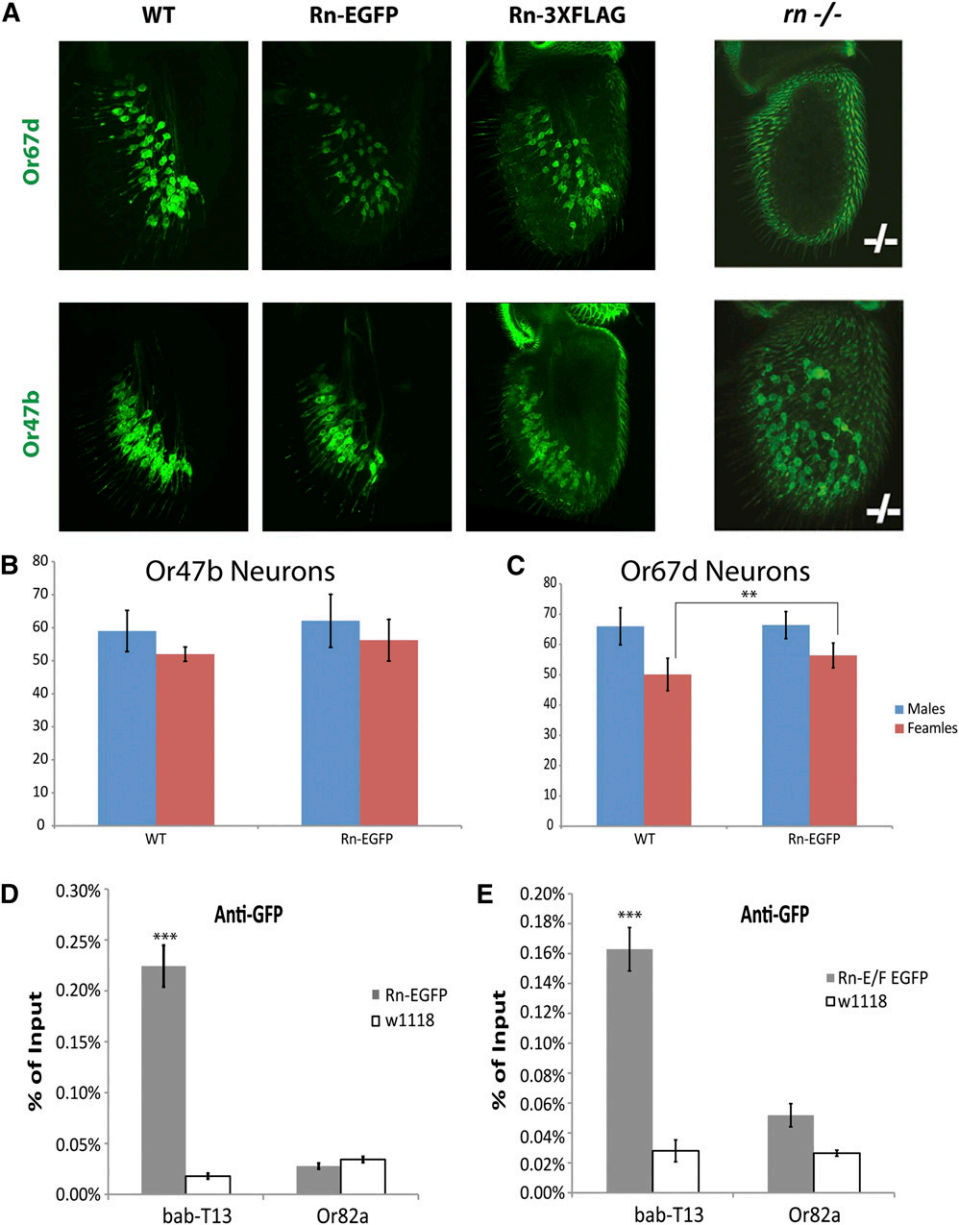
Functional analysis of tagged Rn protein. (A) Flies homozygous for Rn-EGFP and Rn-3X FLAG constructs do not exhibit *rn* mutant phenotypes in the antenna. *rn^tot^* is a previously described allele of *rn*, which is used here as the mutant control. Genotypes from left to right and top to bottom: UAS-mCD8GFP; *Or67d^GAL4^*, UAS-mCD8GFP; *Or67d^GAL4^* Rn-EGFP/Rn-EGFP, UAS-mCD8GFP; *Or67d^GAL4^* Rn-3XFLAG/Rn-3XFLAG, UAS-mCD8GFP; Or67d^GAL4^ FRT *rn^tot^*/*rn^tot^*. UAS-mCD8 GFP *Or47b*-GAL4, UAS-mCD8 GFP *Or47b*-GAL4; Rn-EGFP, UAS-mCD8 GFP *Or47b*-GAL4; Rn-3XFLAG, UAS-mCD8 GFP *Or47b*-GAL4; FRT *rn^tot^*/*rn^tot^*. (B) and (C) Quantification of numbers of neurons from (A). (D) and (E) The *in vivo* ChIP-qPCR analysis shows enrichment for Rn E/F/C isoforms as well as E/F isoforms (E) binding to the previously identified T13 motif upstream of *bric-a-brac2*, but not the M1 motif upstream of *Or82a*. ** *p* < 0.01 *** *p* < 0.001

Mutations in *rn* are associated with a loss of ORN classes generated from *rn*-positive precursors, and an expansion of some *rn*-negative ones (Li *et al.* 2013). For example, in *rn^tot^* mutants, Or67d ORNs, which arise from *rn*-positive precursor lineages, are lost. At the same time, Or47b ORNs from the *rn*-negative lineage are expanded toward the medial region of the antenna (Figure 3A). Both Rn-EGFR and Rn-3XFLAG alleles, when homozygous, displayed the wild-type pattern of Or67d and Or47b ORNs, suggesting that the introduced tags do not interfere with the function of Rn protein (Figure 3A–C). We also did not detect any mutations in the potential off-target sites by sequencing the relevant genomic loci.

Interestingly, both the Rn-EGFP and the RnE/F-EGFP lines are viable. Previous experiments in our laboratory that attempted to rescue the *rn* mutant phenotype by overexpression failed and were able to induce a mutant phenotype in wild-type flies. This discrepancy would suggest that both isoforms are necessary for olfactory system development. Many transcription factors are known to dimerize to properly function, and it is possible that Rn may function in a similar manner, even possibly heterodimerizing the E and F isoforms.

*In vitro* translated Rn protein was previously shown to interact with synthetic regulatory elements of the *bric-a-brac2 (bab2)* gene (Baanannou *et al.* 2013). Both *rn* and *bab2* are expressed in the third instar larval leg and antennal discs (Baanannou *et al.* 2013), and we wanted to test whether this *in vitro* interaction occurs *in vivo*. To test the interaction of the tagged Rn protein with the reported *bab2* regulatory element (T13), we performed chromatin immunoprecipitation from third instar antennal discs followed by qRT-PCR using primers spanning the *bab2* T13 regulatory element. We were able to detect binding of Rn to T13 in the tagged line, but not in the control chromatin extracted from *w^1118^* flies (Figure 3D). As discussed, Rn-E/F isoforms are specifically expressed in the antennal disc. To identify whether the E/F isoforms specifically interact with the *bab2* enhancer, we performed similar experiments using the isoform-specific RnE/F-EGFP line. These studies showed that E/F isoforms also bind to the T13 element upstream of *bab2* (Figure 3E), further supporting the functionality of the tagged protein. As expected, Rn does not bind to the M1 motif in the *rn*-positive *Or82a* promoter *in vivo* (Figure 3, D and E). Together with the nuclear localization, these data provide *in vivo* evidence for and are in agreement with Rn functioning as a transcription factor. The tagged lines will be useful in the future to determine genome-wide binding sites as well as *in vivo* interactors of Rn in the developing olfactory system.

## Supplementary Material

Corrigendum
