# Supplementary material for: Examination of Endogenous Rotund Expression and Function in Developing Drosophila Olfactory System Using CRISPR-Cas9–Mediated Protein Tagging
Source: G3 (Bethesda). 2015 Oct 23;5(12):2809–16. doi: 10.1534/g3.115.021857 (PMC4683652; doi:10.1534/g3.115.021857)
Supplement: Corrigendum [file supp_5_12_2809__index.html]

Corrigendum 

# Examination of Endogenous Rotund Expression and Function in Developing *Drosophila* Olfactory System Using CRISPR-Cas9 Mediated Protein Tagging

## Corrigendum for Li *et al*., *G3* 5 (12): 2809-2816.

**Files in this Data Supplement:**

- Corrigendum - Corrigendum for Li *et al*., *G3* 5 (12): 2809-2816.
