# Supplementary material for: Examination of Endogenous Rotund Expression and Function in Developing Drosophila Olfactory System Using CRISPR-Cas9–Mediated Protein Tagging
Source: G3 (Bethesda). 2015 Oct 23;5(12):2809–16. doi: 10.1534/g3.115.021857 (PMC4683652; doi:10.1534/g3.115.021857)
Supplement: Corrigendum [file supp_g3.115.021857_Li_FINAL.pdf]

Corrigendum for Li *et al.*, *G3* 5 (12): 2809-2816.

*G3*: *Genes* | *Genomes* | *Genetics*, Vol 5, 2809-2816, December 2015, Copyright © 2015 Li *et al*.

CORRIGENDUM

In the article by Q. Li, S. Barish, S. Okuwa, and P. C. Volkan (*G3*: 2809-2816) entitled "Examination of Endogenous Rotund Expression and Function in Developing *Drosophila* Olfactory System Using CRISPR-Cas9 Mediated Protein Tagging," the following sentence has been added to the Acknowledgments section: "This work was supported by funds from Duke University and National Science Foundation to PCV (DEB- 1457690)."
